# The Clinical Utility of Droplet Digital PCR for Profiling Circulating Tumor DNA in Breast Cancer Patients

**DOI:** 10.3390/diagnostics12123042

**Published:** 2022-12-05

**Authors:** Ugur Gezer, Abel J. Bronkhorst, Stefan Holdenrieder

**Affiliations:** 1 Institute of Oncology, Department of Basic Oncology, Istanbul University, Istanbul 34093, Turkey; 2 Munich Biomarker Research Center, Institute of Laboratory Medicine, German Heart Center Munich Technical University Munich, 80636 München, Germany

**Keywords:** breast cancer, cell-free DNA, circulating tumor DNA, mutation, digital PCR

## Abstract

Breast cancer is the most common cancer affecting women worldwide. It is a malignant and heterogeneous disease with distinct molecular subtypes, which has prognostic and predictive implications. Circulating tumor DNA (ctDNA), cell-free fragmented tumor-derived DNA in blood plasma, is an invaluable source of specific cancer-associated mutations and holds great promise for the development of minimally invasive diagnostic tests. Furthermore, serial monitoring of ctDNA over the course of systemic and targeted therapies not only allows unparalleled efficacy assessments but also enables the identification of patients who are at risk of progression or recurrence. Droplet digital PCR (ddPCR) is a powerful technique for the detection and monitoring of ctDNA. Due to its relatively high accuracy, sensitivity, reproducibility, and capacity for absolute quantification, it is increasingly used as a tool for managing cancer patients through liquid biopsies. In this review paper, we gauge the clinical utility of ddPCR as a technique for mutational profiling in breast cancer patients and focus on *HER2*, *PIK3CA*, *ESR1,* and *TP53,* which represent the most frequently mutated genes in breast cancers.

## 1. Introduction

Breast cancer (BCa) is the most common cancer among women, with an estimated 2.3 million new cases globally each year [1]. Between 2005 and 2015, overall incidence increased by 43% because of population growth and aging [2]. BCa will be diagnosed in 12% of all women over their lifetimes and is a leading cause of cancer-related deaths [3]. Mammography is currently the only proven method of BCa screening that reduces mortality, although its accuracy is unsatisfactory in young women with dense breast tissue (sensitivity rates between 25% and 59%) [4].

Breast cancers are categorized into two main histological types: (i) preinvasive in situ cancer and (ii) invasive cancers. In situ cancers account for about 15% to 30% of all cases and are divided into lobular carcinoma in situ and ductal carcinoma in situ. The most common invasive cancer histology is ductal carcinoma, making up to 85% of all cases, followed by invasive lobular carcinoma [5]. Two main molecular markers are determinative in BCa pathogenesis: the first one is estrogen receptor alpha (ERα), which is expressed in up to 70% of invasive breast tumors. Progesterone receptor (PR) is another marker of steroid hormone receptor signaling in BCa pathogenesis. Tumors with at least 1% of tumor cells expressing ERα or PR are categorized as hormone-positive (HR+) [6]. Endocrine therapy aiming to downregulate ERα signaling is the primary systemic therapy for HR+ patients. The second important molecular target in BCa pathogenesis is human epidermal growth factor 2 (*HER2*), a transmembrane receptor tyrosine kinase in the epidermal growth factor receptor family. In approx. 20% of breast tumors, *HER2* is overexpressed; this overexpression is associated with poor prognosis [7]. Patients with *HER2*-overexpressing BCa benefit from anti-*HER2* therapies. Approximately 15% of all breast tumors are triple-negative BCa (TNBC) [8], as they lack the expression of ER, PR, or *HER2.* Patients with triple-negative tumors are at higher risk of distant relapse [9] and have systemic chemotherapy as the only treatment option.

Biomarkers that improve diagnostic, prognostic, and theranostic assessments of BCa are crucial for the management of patients [10]. Moreover, a growing number of patients are requesting personalized therapies, which necessitates the development of new and more versatile biomarkers. Even if the subtyping of breast tumors is mainly based on the immunohistochemical detection of ER, PR, and HER2, this is not sufficient to fully characterize the biology of BCa, which is increasingly recognized as a heterogeneous disease. Gene expression profiling and next-generation sequencing define novel markers with prognostic and predictive values in both early stage and advanced BCa. In recent years, an expanding range of macromolecules are being identified as specific and powerful biomarkers for BCa, such as specific types of circular RNAs, microRNAs, long noncoding RNAs, DNA, proteins, exosomes, and antibodies [11]. Furthermore, the detection of circulating nucleic acids—comprised of segments of genomic DNA and various RNA types, such mRNA and noncoding RNAs—in serum or plasma is increasingly regarded as a potential biomarker in oncology [12]. Although many types of nucleic acids are present in bodily fluids, cell-free DNA (cfDNA) has to date received the most research attention and has demonstrated significant potential as a highly sensitive and specific biomarker for the management of numerous cancer types across all disease stages and phases of cancer management [13]. Although cfDNA is present in many types of bodily fluids, such as serum, plasma, urine, pleural fluid, cerebrospinal fluid, and saliva [14,15,16,17], blood (plasma and serum) represents the most extensively characterized source of cfDNA.

In this review, we explore recent data on the use and potential clinical significance of droplet digital PCR (ddPCR) as a technique for detecting breast cancer mutations in cfDNA from liquid biopsy specimens. To set the context for our appraisal of ddPCR research, we first provide a brief overview of (i) the characteristics cfDNA, (ii) the clinical utility of cfDNA as a biomarker for breast cancer in general, and (iii) sequencing-based mutational profiling of cfDNA.

## 2. Characteristics of Cell-Free DNA

cfDNA represents both single- and double-stranded DNA fragments that are overwhelmingly short, with a size distribution <200 base pairs (bp) [18]. It is released into circulation through active and passive processes by healthy and tumor cells. In healthy subjects, cfDNA is thought to derive mainly from hematopoietic cells undergoing apoptotic cell death [19]. In physiological conditions, the concentration of cfDNA is usually low, in the range of 1–10 ng per mL of plasma. Clearance of cfDNA is rapid (half-life of less than 2.5 h) and occurs primarily in the liver [20]. This short half-life indicates the rapid turnover of cfDNA, consisting of sustained release and clearance. cfDNA is typically fragmented and measures predominantly between 160 and 200 bp [21]. This size distribution corresponds to nucleosome-associated DNA comprising nucleosomes and chromatosomes (nucleosome + linker histone) [22], indicating that a relevant fraction of cfDNA circulates as nucleosomes rather than as free DNA [23]. 

Tumors shed cancer cells, nucleic acids, proteins, and extracellular vesicles into circulation. As a consequence of increased turnover of DNA release, cancer patients generally possess higher levels of cfDNA than healthy individuals, and the proportion of cfDNA derived from the tumor harboring mutations is called circulating tumor DNA (ctDNA) [24]. ctDNA has been detected in many solid tumors with a great variation of its fraction from 0.1% to about 50% [25]. In recent years, the clinical utility of ctDNA saw rapid increase in oncology, possessing broad applicability as an established biomarker in cancer diagnosis and prognosis [25,26]. As a consequence, the approval of the Cobas EGFR mutation test by the FDA to manage therapy in lung cancer with non-small-cell lung carcinoma (NSCL) demonstrates the potential clinical utility of ctDNA in oncology [27].

## 3. The Clinical Value of Circulating Tumor DNA in Breast Cancer

The majority of breast cancers (80–85%) are diagnosed at early stages. As higher cfDNA levels are found in early BCa compared to healthy subjects and those with benign breast lesions and decreased levels of cfDNA after surgical resection of primary tumors (indicating its correlation with tumor burden) [28], ctDNA could be a valuable tool for breast cancer screening. Lin et al. performed a meta-analysis using the findings from 24 eligible studies to predict the diagnostic impact of cfDNA in BCa. Covering and combining different parameters of cfDNA, such as cfDNA quantification, cfDNA integrity, methylated cfDNA, loss of heterozygosity (LOH) etc., the rates of the mean sensitivity, specificity, and area under the curve (AUC) were 0.70, 0.87, and 0.93, respectively, in distinguishing BCa from healthy controls [29]. In another meta-analysis comprised of 13 studies, only cfDNA concentration was utilized. Pooled sensitivity and specificity of cfDNA concentrations were 87% and 87%, respectively, with an AUC of 0.93 [30]. These studies indicate the potential diagnostic value of cfDNA in BCa. However, the lack of tumor specificity of cfDNA concentrations and other parameters hamper its value in screening. Tumor-derived fractions of cfDNA instead of total cfDNA would be an attractive alternative for cancer screening and detection. However, low levels of ctDNA in early BCa are a challenging issue for its use in BCa diagnosis. In order to increase the sensitivity of a ctDNA-based assay, Cohen et al. [31] combined mutated DNA in cfDNA with protein markers and reported sensitivity rates of 43% in stage I, 73% in stage II, and 79% in stage III disease with a specificity of over 99%. In contrast, they detected ctDNA in the majority of patients with metastatic breast cancers. It has been shown that 85.7% of metastatic BCa patients harbored ctDNA compared to 57.8% of non-metastatic patients (stage I–III) [32]. Dawson et al. [33] conducted a comparative analysis for ctDNA, circulating tumor cells (CTCs), and CA15-3. The detection rate of ctDNA was higher (97%) than CTC and CA15-3—with rates of 78% and 87%, respectively—indicating the high diagnostic potential of ctDNA as diagnostic marker in metastatic disease.

As early diagnosis of tumor relapse after a complete primary tumor resection has a high priority in oncological practice, the impact of ctDNA implementation in follow-up has been investigated in several studies. In their prospective study, Garcia-Murillas et al. screened mutations in 14 BCa driver mutations detected in primary breast tumors and found at least one of these mutations in ctDNA in 81% of the patients. The continual presence of mutations in ctDNA 2–4 weeks following surgery was shown to be the most reliable predictor of high risk of early relapse [34]. In the study by Coombes et al., 28 out of 49 patients relapsed. They showed that serial monitoring of ctDNA enabled the detection of metastatic progression with a high sensitivity and specificity before detection by clinical manifestation [35].

Another application of ctDNA in BCa is predicting the pathologically complete response (pCR) during neoadjuvant chemotherapy (NAC), as ctDNA is considered a sensitive method for evaluating treatment response in the neoadjuvant setting. Magbanua et al. assessed the use of longitudinal ctDNA measurements to predict pCR and risk of recurrence via ultra-deep sequencing of 16 patient-specific mutations, which were assessed before starting and 3 weeks following the start of paclitaxel therapy, between paclitaxel and anthracycline regimens, and prior to surgery. They showed that ctDNA clearance during the treatment course correlated with greater survival rates—even in patients who did not achieve pCR—while the lack of ctDNA clearance correlated strongly with poor response and metastatic recurrence, indicating the potential of ctDNA for real-time assessment of treatment response during NAC in BCa patients [36].

As demonstrated by these studies, ctDNA profiling offers a broad spectrum of applications in BCa. In addition to high detection rate in metastatic patients, ctDNA represents an attractive, non-invasive tool to monitor tumor evolution and treatment response, and assess prognosis [37]. Serial monitoring of ctDNA could help to predict relapse in previously treated patients and to select the best therapy in individualized treatment which will help to improve the management of cancer patients.

## 4. Methods for the Detection of Circulating Tumor DNA

### 4.1. Sequencing-Based Techniques

As mutated DNA molecules constitute a tiny fraction of the total cfDNA population, the differentiation between tumor-specific alterations and their wild-type counterparts is of high importance. Mutations in cfDNA can be detected either by PCR or sequencing. Sanger sequencing was the first technique employed to detect ctDNA in plasma. However, it has not been a feasible approach due to many shortcomings, such as low-throughput, strenuous protocols, and high cost [38]. In the 2000s, next-generation sequencing (NGS) technology with many effective and convenient sequencing approaches superseded Sanger sequencing. Mutation analysis in plasma via NGS can be targeted or untargeted [39]. In the untargeted approach of whole genome sequencing, no enrichment of target sequences is performed. Even if the sequencing depth is impaired in whole-genome sequencing (WGS), it can enable the discovery of new genetic alterations relevant to prognosis of patients. For tumor mutation analysis, an accurate identification of ctDNA with a high background cfDNA is a key issue [40]. The terms mutant allele frequency (MAF) and variant allele frequency (VAF) are usually employed to assess the performance of a ctDNA profiling assay [41]. This mutational frequency describes the number of sequencing reads that contain the mutant allele divided by the total number of sequencing reads. Lower MAF rates are indicative of a more sensitive assay for ctDNA analysis. Many issues, such as low sensitivity in early-stage cancer [40] and high costs, limit the use of NGS-based methods in clinical practice. WGS studies of cfDNA in BCa go back to the early 2010s [33,42]. In one of the first studies, Dawson et al. identified somatic genomic alterations in 97% of patients, showing good correlation with changes in tumor burden [33]. In a more recent study using high coverage WGS for mutational analysis of cfDNA, the mutation content of the plasma samples was found to be higher than in the matched breast tumor samples. Interestingly, 90% of somatic cfDNA alterations were not detected in matched tumor tissues and were due to two background mutational signatures. Intriguingly, cfDNA fragments ranging from 300 bp to 350 bp in size (e.g., di-nucleosomal plasma DNA) had a much higher proportion (30%) of mutations in common with the tumor [43]. The findings of this study show the complexity of ctDNA analysis using WGS.

In contrast to WGS, in targeted ctDNA profiling, a panel of genes or whole exosomes are screened for cancer-specific alterations. As targeted sequencing is relatively low-throughput, clinically relevant mutations can be detected with high sensitivity by deep sequencing. Due to increased specificity and sensitivity, targeted sequencing is considered to be more applicable for clinical diagnosis [44]. During library construction target enrichment is achieved by direct PCR amplification or hybridization capture of target sequences. In multiplex PCR-based methods, the length of the fragments is a crucial parameter and several reactions are often simultaneously run for target enrichment in order to cover a broader range of the genome. In hybrid capture methods, complementary RNA probes which can detect both single nucleotide alterations and structural variants are employed [45]. It is noteworthy that the extent of cfDNA as a source of heterogeneity can affect coverage across targeted exons, which should be taken into consideration when designing the assays for targeted ctDNA sequencing [46].

Several targeted sequencing techniques or protocols have been used in ctDNA analysis. Some of them are briefly discussed below. The so-called Safe-Sequencing System (Safe-SeqS) was the first technique to apply molecular barcodes in DNA sequencing, which increased the sensitivity of NGS [47]. In this approach, a unique barcode is defined to each template molecule, and PCR amplicons with the same unique identifier are regarded as mutant if more than 95% of them contain the identical alteration. This strategy is said to reduce the sequencing errors substantially and was associated with a high sensitivity rate (~98%) for detecting cancer-specific mutations [47]. The tagged amplicon deep sequencing (TAm-Seq) technique developed by Forshew et al. [48] is intended to sequence large genomic regions. In this approach, a targeted preamplification step precedes the amplification of target regions. Finally, in a further PCR, specific barcodes and adaptors are linked to amplicons. This assay harbored a high rate (>97%) of sensitivity and specificity. Duplex sequencing is an advanced approach of the Safe-SeqS technique [49], in which a semi-degenerated unique barcode is ligated to target DNA. Following sequencing, after comparing the sequences with the duplex adaptors, mutations are retained only if there is a consensus between both strands. Several studies including various cancer types applied this technique, and a MAF of 0.1% and below with high sensitivity and specificity was reported [50,51]. Cancer personalized profiling by deep sequencing (CAPP-Seq) is a technique combining target enrichment via hybrid capture with deep sequencing with the goal of improving the sensitivity of mutation detection [38]. It generates a library of selectors (e.g., biotinylated oligonucleotide probes) that specifically bind to mutated genomic sequences for target enrichment. Simultaneous detection of various types of alterations, such as single nucleotide variants, rearrangements, insertions/deletions, and copy number variations, represents an advantage for improved sensitivity of the assay. It has been possible to identify mutations in patients with stage II–IV NSCLC with 96% specificity and a low mutant allele fraction of 0.02% [38].

Numerous studies investigated the mutation profile of cfDNA in BCa using targeted sequencing. In the plasmaMATCH trial, in which plasma DNAs were sequenced by duplex error corrected sequencing covering a 74-cancer-gene panel, 92.9% of 800 patients with advanced BCa had at least one ctDNA alteration. The genes with highest rates of mutations were *TP53* (44.1%), *PIK3CA* (34.9%), *ESR1* (33.1%), *GATA3* (11.0%), *ARID1A* (7.8%), and *PTEN* (6.9%) [52]. The Oncomine Breast Cancer cfDNA assay (Thermofisher) is an often-used, commercially available targeted sequencing assay covering 152 hotspots in several driver genes, including *PIK3CA*, *ESR1*, *TP53*, *EGFR*, *HER2,* and *KRAS,* and copy number variations in CCND1, HER2, and FGFR1 genes. Shibayama et al. used this assay to determine the mutation spectrum of ctDNA among HR+ mBCa. Among 56 patients, *PIK3CA*, *TP53,* and *ESR1* were the most mutated genes, with frequencies of 51.7%, 30.3%, and 16.0%, respectively. Patients positive for ESR1 mutations harbored shorter progression-free survival (PFS) than those without mutations. Thus, the assessment of ESR1 mutations could be a useful tool in predicting prognosis of patients with HR+ metastatic BCa [53]. In the work of Shim H et al. utilizing this assay, at least one ctDNA alteration was detected in 82% of BCa patients, with the most common mutations detected in *TP53* (50%), *PIK3CA* (15%), and *ESR1* (14%). In serial monitoring of variants detected, changes in the allele frequency of *PIK3CA* and *TP53* mutations were reflective of response to chemotherapy [54]. In a report with 373 women including healthy controls (N = 127), ductal carcinoma in situ (N = 28), primary breast cancers (N = 60), primary breast cancer on follow-up (N = 47), and metastatic breast cancers (N = 111) using the Oncomine Breast Cancer cfDNA assay, *ESR1*, *TP53*, and *PIK3CA* mutations accounted for 93% of all variants detected and predicted poor overall survival (OS) in mBCa [55].

### 4.2. PCR-Based Methods

PCR-based methods, such as quantitative PCR (qPCR) and digital PCR, are rapid, relatively inexpensive, and have a rather simple workflow. They enable high-specificity detection of single mutations at very low MAFs (0.1% and lower) [56]. Probe-based qPCR is based on the measurement of the fluorescence emitted following the hydrolysis of target-specific probes during amplification, while the amount of target DNA is determined relative to a standard curve generated with a sample of known copy number or quantity. As a widely recognized gold standard, qPCR is the most widely used method with its simple workflow and relatively robust results. Subsequently, qPCR assays were developed to detect tumor-specific mutations in the cfDNA fraction, and the sensitivity of mutation detection was increased by selective amplification of mutant alleles. Below we briefly introduce some specific qPCR techniques developed for mutation detection. 

ARMS-PCR (amplification-refractory mutation system PCR) enables the detection of point mutations by allele specific primers. In this technique, the mismatch at the 3′ end of the primer substantially reduces the annealing and hence the amplification. This feature is achieved by the lack of 3′ to 5′ exonuclease proofreading activity of the Taq polymerase. The limit of detection of this assay has been variable across different studies, with relatively high false positive rates with a detection limit around 0.5 to 1% in plasma DNA [57,58]. Another technique, PNA-LNA (peptide-nucleic-acid-locked nucleic acid) Clamp PCR, employs peptide nucleic acids as clamps, preventing the amplification of wild-type DNA to favor the selection of variant alleles. Thiede et al. [59] were the first group to detect mutations in *KRAS* using PNA clamping. A PNA clamp specifically designed for the wild-type *KRAS* gene allowed selective amplification of clinically relevant mutations of codons 12 and 13. This technique has also been applied to the detection of epidermal growth factor receptor gene (*EGFR*) mutations, especially T790M mutations in tumors resistant to EGFR-TKIs (tyrosine kinase inhibitors). The sensitivity and specificity of the detection of a sensitizing *EGFR* mutation were found to be 72.7% and 100%, respectively, in advanced NSCLC and 0 and 100%, respectively, in early-stage NSCLC [60]. COLD-PCR (co-amplification at lower denaturation temperature PCR) is a single-step amplification method that results in the enrichment of wild-type and low-abundance variant alleles during PCR. Differentiation of variant alleles occurs by exploiting the critical denaturation temperature at which a mutant allele is preferentially melted over a wild type. COLD-PCR has been used to improve the sensitivity of a number of subsequent assays that traditionally include conventional PCR such as pyrosequencing or Sanger sequencing [61].

## 5. Digital PCR Technology

Even if qPCR is a powerful tool for the detection and quantification of tiny amounts of DNA/RNA molecules, it is associated with many shortcomings. In qPCR, it is assumed that the sample and the standards have equivalent amplification efficiencies. However, any changes in PCR efficiencies can markedly alter the accuracy of quantification [62,63]. Additionally, low tolerance to interfering substances in PCR reactions may affect the outcome of measurements [64]. Despite these drawbacks, qPCR has been widely used in clinical settings as the main method of nucleic acid quantification [65].

**Figure 1:**
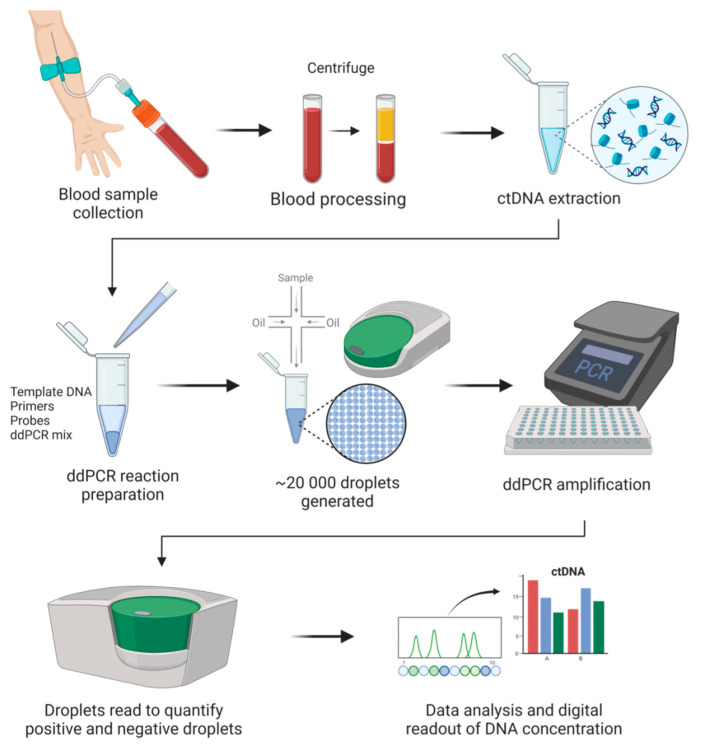
General digital droplet PCR (ddPCR) workflow in cancer liquid biopsies.

Since the initial concept validation in the early 1990s [66], digital PCR technology has been increasingly used in nucleic acid quantification. In many cases, digital PCR acts as a supplement to NGS. Digital PCR’s advantages have made it applicable in many fields, primarily in precision medicine and clinical diagnostics (e.g., liquid biopsy, prenatal testing). Like many other techniques, multiple platforms have been developed, and currently, several commercialized digital PCR platforms are available. Based on the partitioning techniques, there are two main types of commercial digital PCR platforms: (1) chamber-based digital PCR (cdPCR) and (2) droplet digital PCR (ddPCR). cdPCR, also known as chip-based digital PCR or microwell digital PCR, performs passive partitioning to create subvolumes and features microchamber arrays containing a PCR mixture to target nucleic acids to be amplified [67]. Commercial cdPCR devices include the BioMark series (Fluidigm Corporation), the QuantStudio series (Thermo Fisher), Constellation/QIAcuity (Formulatrix), and Clarity (JN MedSys). ddPCR (also known as emulsion digital PCR) partitions samples by use of water-in-oil emulsions [68]. A crucial component of this technique is the surfactant and oil formulation, which assures the stability of droplets and their compatibility with molecular reactions (e.g., PCR). This technique is capable of manipulating small partition volumes, allows control over size dispersity, and enables continuous flow for droplet production. Additionally, low consumption of reagents represents another advantage offered by this technology. Microfluidic droplet-based strategies are used by the following platforms: the QX series (Bio-Rad), RainDrop (Bio-Rad), Naica (Stilla Technologies), and BEAMing (Sysmex). As of 2022, the four most notable commercial brands are already in the digital PCR market [69]. These are the QX series, RainDrop, the BioMark series, and the QuantStudio series. In a recent review, various available platforms were compared in terms of accuracy and range, user convenience, throughput, sample recovery, detection channels and multiplexing, contamination risk, and cost [69]. Dong et al. compared four ddPCR platforms (QX100, RainDrop, Biomark, and QuantStudio 12k), all of which showed comparable efficiencies in determining the copy number of DNA [70]. ddPCR is currently the most commonly used digital PCR approach for the analysis of ctDNA (Figure 1).

DNA quantification by ddPCR is based on limiting dilutions, PCR, and Poisson distribution. As the next generation of PCR technology, ddPCR enables absolute quantification of target molecules in a sample by partitioning the reaction with high precision and accuracy. In ddPCR, template molecules in a reaction are first randomly partitioned into a large number (~20,000) of separate PCR sub-reactions, in which each partition or droplet receives either no template or one or more copies. Amplification occurs in those partitions containing any number of target molecules (positives), and no amplification is detected in those partitions with zero copies of target molecules (negatives). After PCR, the number of droplets with negatives and positives is counted based on the fluorescence amplitude. Sample partitioning results in the enrichment of target molecules within the isolated drops, and this enrichment effect limits template competition. In this way, the detection of rare variants in the presence of high background of wild-type sequences is enabled. The concentration of the target nucleic acid is calculated by Poisson statistical analysis without the need for a standard curve [71]. Due to the inherent stochasticity of partitioning, the fluorescence signals produced during amplification generate a Poisson distribution. The fraction of amplification-positive partitions is used to calculate the concentration of the target sequence or variant allele. ddPCR has many applications, mainly mutation detection and copy number variation [72]. ddPCR offers many advantages, e.g., greater precision and sensitivity for the detection of low copy variants and high tolerance to inhibitors [65,73]. More importantly, the ability of ddPCR to facilitate a calibration-free quantification represents a superior advantage over qPCR [74].

## 6. Effects of Preanalytical Variables on Digital PCR

One of the major limiting factors in ctDNA analysis is that tumor-derived DNA represents only a small fraction of the total amount of cfDNA (from <0.01% to >90%), hampering its clinical utility [25]. Thus, the low fraction of mutated DNA sequences in blood circulation necessitates large input volumes of plasma to allow sufficient copy numbers, especially in early breast cancer. In addition to the scarcity of ctDNA, the sensitivity of methodologies for mutation detection and quantification [75,76], the efficiency of extraction methods, and many preanalytical variables—including blood collection, centrifugation, and storage conditions [77,78,79]—lead to interstudy differences and affect the reliability of ctDNA testing. Considering the isolation of cfDNA, various commercially available kits were compared in several studies for their efficiencies. Bead-based isolation methods were slightly better than membrane-based kits and were shown to be especially suitable for the extraction of low-molecular-weight DNA [80]. Preanalytical variables can also affect the release of background DNA from blood cells and lead to the dilution of the ctDNA proportion. As digital PCR results on cfDNA could be considerably affected by multiple variables during preanalytical sample workup, they should be addressed when developing diagnostic tools in oncology [79,81].

A number of studies assessed the effect of preanalytical variables on the performance and reproducibility of ddPCR in plasma samples from patients with breast and other cancer types. Hrebien et al. evaluated the reproducibility of ddPCR in paired blood samples, which were processed either immediately or in 48–72 h after collection. They analyzed plasma DNA with multiplex ddPCR assays for hotspot mutations in *PIK3CA*, *ESR1,* and *HER2* and for *AKT1* E17K in 96 paired samples from patients with advanced BCa. A concordance rate of 95% was detected in mutation calling between samples that were processed immediately and with a delay. They concluded that delayed processing of plasma samples using preservative tubes do not significantly affect ctDNA mutation analysis by ddPCR [82].

In the work by Cavallone et al., the effect of many preanalytical variables, such as DNA extraction protocol, timing and speed of the second centrifugation, and the use of blood collection tubes (k-EDTA and CTAD) on the results of ctDNA analysis in BCa has been assessed. By targeting mutations in *TP53*, *CDH5, MAP1LC3B*, *ROBO2,* and *PARK2* genes using ddPCR, they showed that an in-house hybrid protocol produced significantly higher cfDNA concentration than the commercial kits. ctDNA concentration was not significantly affected by the type of blood collection tube. Additionally, the speed of second centrifugation and storing plasma samples up to two weeks at −80°C before the second spin did not alter cfDNA levels [83].

One of the crucial issues in the field of liquid biopsy is the source of cfDNA for mutational analysis. Plasma and serum cfDNA are available sources of tumor DNA. In their study, Takeshita T et al. compared plasma and serum for the ctDNA content using hotspot mutations in *ESR1* and *PIK3CA* in ER+ mBCa patients through ddPCR. Among 33 patients, the mutation frequency of *ESR1* in plasma was higher than in serum. *PIK3CA* exon 9 and exon 20 mutations were detectable in 10 out of the 33 patients in plasma. In sera, *PIK3CA* hotspot mutations were detected in 5 out of 10 with *PIK3CA* mutations in plasma. The authors concluded that plasma samples should be considered the preferred source of cfDNA [84].

## 7. Use of Digital PCR in ctDNA Analysis in Breast Cancer Patients

The first use of ddPCR in serial monitoring of BCa patients goes back to the early 2010s. Olsson et al. combined low-coverage WGS in primary tumors with ddPCR to quantify tumor-specific alterations and showed that ctDNA monitoring is highly accurate to postsurgically discriminate patients with recurrence from those without recurrence. Mutation detection was shown to precede clinical detection of metastasis in 86% of patients, whereas patients with long-term survival had no detectable ctDNA postoperatively. High ctDNA levels were predictive of poor survival. These findings revealed the potential of ddPCR-based ctDNA monitoring for early metastasis detection and therapy modification [85]. In line with these findings, the impact of ctDNA analysis on clinical management of mBCa patients was also evidenced by Bujak et al. who used hotspot mutations in several genes (*PIK3CA*, *ESR1*, *HER2*, and *AKT1*) detected by multiplexed ddPCR and targeted NGS (in a subset of samples) at baseline as well as serial plasma testing during the course of patient monitoring. Using ddPCR and targeted panel sequencing it was possible to identify at least one mutation at baseline in 80 out of 234 patients and in 62 out of 159 of patients, respectively. Increased ctDNA levels were associated with poor OS [86].

The response to NAC in TNBC is associated with favorable prognosis and determines the need for adjuvant chemotherapy. Cavallone et al. evaluated the clinical value of ctDNA trough serial analysis of plasma samples prior, during, and after NAC. Individual ddPCR assays were developed for 121 different mutations. They found that ctDNA levels drastically declined after a single cycle of NAC, especially in patients who had pCR whereas in cases with significant residual tumor at surgery, ctDNA levels rose. The detection of ctDNA at early stages of treatment and also at the end of NAC before surgery was shown to be strongly predictive of residual tumor at surgery. Mutation detection at the end of NAC was predictive of poor relapse-free survival and OS. The results of this study reveal that individualized ctDNA profiling during and after NAC has predictive and prognostic value in early-stage TNBC patients [87]. 

A further application of ddPCR other than the detection of point mutations is the analysis of copy number variation (CNV) in ctDNA, as exemplified in the *CCND1* gene. Cyclin D1 protein encoded by *CCND1* is involved in the regulation of G1 to S phase transition in the cell cycle. *CCND1* amplification is a common event in BCa and often results in increased cyclin D1 expression [88]. This alteration has also been found to be associated with positive receptor expression and luminal subtypes and is a prognostic factor for recurrence-free survival (RFS) and OS [88,89]. A recent study by Shimazaki et al. [90] analyzed *CCND1* amplification in ctDNA of BCa patients with luminal B subtype using ddPCR. *CCND1* alteration was detected in 16% of cases, and patients with *CCND1* CNV positivity had significantly shorter recurrence-free survival than those without *CCND1* amplification.

Given the potential clinical value promised by ctDNA assessments, we will review recently published data on the use of ddPCR-based characterization of ctDNA—in particular, the assessment of *HER2* amplification and *PIK3CA*, *ESR1*, and *TP53* mutations for the monitoring of BCa patients during targeted therapies and the management of BCa patients in general. 

### 7.1. Detection of HER2 Amplification in Plasma via Digital PCR

HER2 is a member of the ErbB family of receptor tyrosine kinases and is encoded by the *HER2* gene located on human chromosome 17q21. In 15–20% of invasive breast cancers, the *HER2* gene is amplified and the *HER2* protein is overexpressed [91]. *HER2* overexpression is a poor prognostic factor with high rates of recurrence and mortality [91]. Importantly, it is the only predictive marker that informs on the benefit of HER2-targeted therapy, which is exclusively effective in HER2-overexpressed breast cancers. Thus, the accurate determination of HER2 status is a vital milestone towards the treatment of BCa. Currently, the detection of HER2 overexpression by immunohistochemistry (IHC) and the amplification of *HER2* by fluorescence in situ hybridization (FISH) are the two main standard methods used to detect HER2 status in clinical practice [92]. Although there is a high concordance between IHC and FISH techniques as standard methods in clinical practice [93], many laboratories across the world use IHC as a screening test and FISH as a confirmation test [94]. IHC is generally preferred over FISH due to the relatively high failure rate, extended protocol duration, and high costs of FISH vs. IHC [94]. Nevertheless, even IHC is not fully unproblematic, as proteins can be easily damaged in formalin-fixed and paraffin-embedded (FFPE) tissues during fixation or any other processing. Additionally, differences may exist among antibody batches, and the judgement of the staining results is subjective [95]. Furthermore, some biological issues, such as the spatiotemporal heterogeneity of HER2 within tumors, neoadjuvant chemotherapy, relapse, and metastatic progression, may hamper the accurate determinations of the true HER2 status of patients. In addition, novel mutations that were not present in the first biopsy may emerge over the course of cancer progression [96]. As repeated tissue biopsies are not typically performed in standard clinical practice, these temporal fluctuations in the mutational landscape of tumors are often overlooked and remain undetected. Therefore, supplementing traditional tissue biopsy with additional information may help overcome some limitations of HER2 testing in tissue specimens. Here, the determination of HER2 status in the plasma of BCa patients offers an alternative approach. Many early studies used qPCR to assess HER2 status in cfDNA [97,98]. Using targeted sequencing, several studies demonstrated the feasibility of HER2 testing in ctDNA for therapeutic response monitoring in BCa patients [99,100].

Depending on the amplification level of *HER2,* its copy number can range from 4 to 8 copies to 20+ copies [101]. Similarly, at a tumor content of 70% in the tissue, the expected ratio of the digital PCR tests can vary from 2.8 to over 14 [101]. However, when the assays are applied on plasma ctDNA analysis, the expected ratio will be determined by two factors: ctDNA level in plasma determined by tumor burden and the extent of *HER2* amplification. Gevensleben et al. [102] were the first group to adapt a ddPCR assay to determine the oncogenic amplification of *HER2* in the plasma of metastatic breast cancer (mBCa) patients. Using *EFTUD2* at chromosome position 17q21.31 as the reference gene, the HER2:EFTUD2 ratio had an AUC of 0.92 in the development cohort at a cutoff value of 1.25. In the validation cohort, 64% of patients with HER2-amplified cancers were plasma-HER2-positive, whereas 94% of patients with no HER2 amplification in tumors had no ddPCR-based HER2 amplification in their plasma. Zhou et al. [101] demonstrated that the coefficient of variation of HER2 ctDNA ranged between 2–3%, while 2.36 copies per diploid gene was the limit of detection. With their validated HER2 assay being highly concordant with tissue biopsy results (i.e., IHC, FISH), they performed a 6-month longitudinal study to assess the feasibility of plasma HER2 testing during targeted therapy with ddPCR in a stage IV invasive ductal carcinoma patient and demonstrated the usefulness of the assessment of *HER2* amplification status by ddPCR in ctDNA as a monitoring tool during targeted treatment.

Using a ddPCR assay, Xie et al. [103] also evaluated the HER2 status in the ctDNA of 224 BCa patients. Paired plasma HER2 testing was compared to levels of HER2 in tissues. An overall concordance level of 66.96% between plasma ddPCR and IHC/FISH tissue testing was reached. The sensitivity between ddPCR with cfDNA and tissue IHC/FISH was found to be 43.75% (42/96), and the specificity was found to be 84.38% (108/128). When stage III, stage IV, and recurrent/mBCa were separately evaluated and compared with tissue IHC/FISH HER2 measurements, the sensitivity of dPCR-based ctDNA detection increased considerably for stage III and IV. In concordance with increased sensitivity at higher tumor burden, recurrent BCa patients had an increased sensitivity of 51.61% (16/31). Interestingly, recurrent BCa patients had lower specificity (67.86%). The authors interpreted this finding, in part, as a consequence of inter- and intratumor heterogeneity that was missed by tissue biopsies but detected by ctDNA analysis. For late-stage or relapsed BCa patients or those who became resistant to therapy, ctDNA-based mutational profiling may be performed as a follow-up test after negative tissue biopsy results to minimize false-negative diagnoses. The studies summarized above reveal the need of liquid biopsy for HER2 amplification and demonstrates the potential of ddPCR to serve as a useful method in the companion diagnostics toolbox.

### 7.2. ddPCR-Based Detection and Clinical Use of Circulating PIK3CA Mutations in Breast Cancer

The PI3K/AKT/mTOR is a complex signaling pathway with essential effects on cellular activities, such as cell proliferation, cell metabolism, apoptosis, and angiogenesis [104]. Upon activation, the receptor activates PI3K (phosphatidylinositol (3,4,5)-trisphosphate kinase). Phosphorylation of PIP2 by activated PI3K generates PIP3, which recruits AKT and PDK1 kinases to the cell membrane, where AKT is phosphorylated by mTOR complex 2, which leads to a change in the conformation of the AKT. The activated AKT first phosphorylates its target proteins on the cell membrane, and after loss of its connection with the cell membrane, it phosphorylates other targets in the cytosol and nucleus. The phosphorylation of target proteins induces cell proliferation and survival [104]. Mutations or amplifications in the catalytic subunits p110α (PIK3CA) and p110β (PIK3CB) in BCa affect the PI3K pathway [105]. The alpha isoform of PI3K (p110α) is hyperactivated by the P*IK3CA*-activating mutation. Frequently mutated in human cancer, *PIK3CA* mutations are detected in 30–40% of patients with BCa [106] and in approximately 40% of tumors that are HR+/HER2− [107]. The most *PIK3CA* mutations are located in two hotspot regions, i.e., exon 20 (H1047R or H1047L) and exon 9 (E542K or E545K) [108]. Despite controversial prognostic value, the predictive impact of *PIK3CA* mutations as a surrogate marker for the molecular response of a tumor to therapeutic inhibition of the PI3K pathway is now well understood [109]. 

The PI3K/AKT/mTOR pathway represents one of the major mechanisms involved in the resistance to endocrine therapy. Therefore, assessing or tracking of its mutations in blood is of high clinical relevance. In this context. many studies analyzed *PIK3CA* (Table 1) or *AKT* mutations in the plasma of treatment-naïve breast cancer patients or in relation to hormone or targeted therapy. Sato et al. elucidated the clinical significance of the *PIK*3*CA* mutations in early-stage BCa. The *PIK*3*CA* mutations were detected in 48% of primary tumors. Of 12 cases with the mutated *PIK*3*CA* in a tumor, 4 showed the identical mutation in pre-surgery plasma. Furthermore, *PIK*3*CA* mutation detection in pre-surgery plasma samples was a predictive indicator of tumor burden and prognosis [110]. In a cohort with HR+/HER2 negative mBCa patients, hotspot *PIK3CA* mutations (exons 9 and 20 and N345K, C420R) were screened using ddPCR. Of 89 patients, 32% had at least one *PIK3CA* mutation in cfDNA, and an agreement of 83% between the ctDNA and the corresponding tumors was achieved [111]. Nakai et al. also analyzed exon 9 and 20 *PIK3CA* mutations in mBCa patients using ddPCR. Of the 52 patients recruited, 13 had *PIK3CA* mutations in tumor tissue, and *PIK3CA* mutations in plasma were detected in 15% of the patients. The sensitivity for detecting ctDNA *PIK3CA* mutations was 31% [112]. Allouchery et al. investigated *PIK3CA* mutations in inflammatory BCa, an aggressive subtype with poor outcome. *PIK3CA* mutations were detected in 14/55 patients (25%) in tumors at baseline biopsy, and corresponding circulating *PIK3CA* mutations were found in 55% of those patients with sufficient plasma DNA for ctDNA analysis [113]. In the study by Dumbrava et al., in a cohort of 68 patients with breast (n = 41) and colorectal cancer and some other tumor types, the prognostic value of *PIK3CA* mutations was assessed using ddPCR. Most patients (85%) had mutated *PIK3CA* genes in tumor tissue, and 74% had *PIK3CA* mutations in plasma DNA, with a concordance of 72%. Patients with a higher VAF had shorter median survival compared to those with a VAF of ≤8.5% (15.9 vs. 9.4 months). Serial analysis of ctDNA showed that patients with a decrease in *PIK3CA* mutation fraction had a longer time (10.7 months) to treatment failure compared to an increase or no change (2.6 months) [114]. In a cohort of TNBC patients who relapsed after surgical resection, *PIK3CA* (H1047R) and *AKT1* (E17K) mutations were common in surgically resected samples. These predefined mutations were also detected in plasma by ddPCR [115].

Serial monitoring of tumor-derived cfDNA is increasingly used to assess the response to anticancer therapies and detect minimal residual disease (MRD). Wood-Bouwens et al. developed a single-color ddPCR assay for the detection and quantification of specific mutations, including *PIK3CA* in ctDNA, and tested it for the long-term monitoring of patients with metastatic cancer, including BCa. ctDNA levels were shown to correlate with serum tumor markers (such as carcinoembryonic antigen, CA-19-9, and CA-15-3) of metastatic cancer burden and qualitatively corresponded to imaging data. Similar results were also obtained among patients under active treatment, demonstrating that personalized ctDNA analysis with a longitudinal monitoring may be a useful predictor of treatment response in metastatic cancer [116]. Jacot W et al. evaluated the prognostic significance of ctDNA-based *PIK3CA* mutation detection via ddPCR in first-line-hormone-therapy-treated mBCa patients. Blood samples were collected before and 4 weeks, 3 months, and 6 months after starting hormone therapy as well as at tumor progression. Most patients (87%) were treated with an aromatase inhibitor (AI). *PIK3CA* mutations were detectable in 28% and 14% of women at baseline and 4 weeks after therapy initiation, respectively. The persistence of detectable circulating *PIK3CA* mutation at 4 weeks was associated with a shorter PFS at 1 year (40% vs. 77%) [117]. Darrigues et al. evaluated the impact of early changes of ctDNA levels (e.g., *PIK3CA*, *AKT1,* and *TP53* mutations) during a therapy course with palbociclib (a CDK4/6 inhibitor) plus fulvestrant (a selective estrogen receptor degrader) on efficiency in patients with ER+/HER2-negative mBCa. ddPCR-based mutation analysis was performed in plasma samples at baseline and at days 15 and 30. Of 61 patients enrolled, 21 (34%) had *PIK3CA* mutations, whereas *AKT1* and *TP53* mutations were rare (each 3%). Baseline levels of mutated cfDNA had no prognostic value. Among patients with mutations, ctDNA was still detectable in 82% at day 15 and 68% at day 30. ctDNA clearance at day 30 was associated with favorable PFS (HR = 7.2), and those patients with higher mutation levels at day 30 than at baseline had a shorter PFS (HR = 5.1). Patients with increases in ctDNA levels at day 30 experienced disease progression after 3 months under treatment with palbociclib–fulvestrant. Furthermore, ctDNA was also detected in all patients tested for radiological tumor progression [118]. The authors concluded that serial ctDNA analysis enables the monitoring of the efficacy of palbociclib and fulvestrant before radiological evaluation and that early variations in ctDNA levels may be useful to assess prognosis of patients.

In the BEECH study in which paclitaxel plus placebo was compared versus paclitaxel plus AKT inhibitor capivasertib in patients with ER+ advanced mBCa (ER+ mBCa), Hrebien et al. assessed the clinical value of ctDNA as a surrogate for PFS and drug efficiency [119]. cfDNA at baseline and at multiple time points in the test cohort and validation cohort was utilized for longitudinal analysis and hotspot mutations, including *PIK3CA* using ddPCR. The primary goal was to assess the impact of early clearance of ctDNA for outcome prediction in the validation cohort. In the development cohort, the absence of ctDNA in plasma was obvious after 8 days of treatment, and the optimal time point to predict PFS was cycle 2 day 1 (4 weeks). In the validation cohort, median PFS was found to be 11.1 months in patients with cleared ctDNA at 4 weeks, whereas PFS was 6.4 months in patients with detectable ctDNA. These findings reveal that ctDNA analysis at early stages may serve as a surrogate marker of PFS [119].

**Table 1:** An overview on the reports which analyzed circulating PIK3CA mutations in breast cancer patients using droplet digital PCR.

Reference	PIK3CA Mutations Analyzed	Study Cohort	Goal of the Study	Main Finding
Sato et al., 2021 [110]	E542K, E545K, H1047R	Early-stage breast cancer	Assessing the significance of ctDNA in early-stage breast cancer.	*PIK3CA* mutation in in plasma is detectable in a subset of patients. Pre-surgery ctDNA is a useful predictive indicator of tumor burden and prognosis.
Corne et al., 2021 [111]	E542K, E545K, H1047R, H1047L. N345K, C420R	HR+/HER2 negative metastatic breast cancer	To detect the frequency and quantify *PIK3CA* mutations.	More than a third of patients had at least one mutation in their plasma, and high agreement between ctDNA and corresponding tumors.
Nakai M et al., 2022 [112]	E542K, E545K, H179R H1047R, H1047L.	Metastatic breast cancer	To detect the frequency of *PIK3CA* mutations.	*PIK3CA* mutations were detected in 15% of patients. In some patients with *PIK3CA* mutations in plasma, no PIK3CA mutations were detected in the primary tumors.
Allouchery et al., 2021 [113]	E542K, E545K, H1047R H1047L	Locally advanced inflammatory breast cancer	Evaluating the detection rate of circulating *PIK3CA* mutations on initial biopsy.	25% of the patients had a *PIK3CA* mutation in tumor at baseline. *PIK3CA* mutations in cfDNA were found in 55% of those with enough plasma DNA for ctDNA analysis.
Dumbrava et al., 2021 [114]	E542K, E545K, H1047R, H1047L and AKT1 (E17K) mutations	Advanced breast cancer	Evaluating prognostic value of circulating *PIK3CA* mutations.	Patients with a higher mutation frequency had shorter survival, and a decrease in VAF was associated with a longer time to treatment failure.
Okazaki et al., 2021 [115]	H1047R	Triple-negative breast cancer	Detection of *PIK3CA* mutations in tumor and plasma in patients who relapsed after surgical resection.	Retrospective detection of *PIK3CA* mutations is applicable to cfDNA in relapsed patients.
Wood-Bouwens et al., 2020 [116]	H1047R	Metastatic cancer	Evaluating the value of personalized ctDNA analysis for monitoring patients with metastatic cancer.	ctDNA levels correlated with serum markers of metastatic burden. Personalized ctDNA analysis with a longitudinal monitoring is a useful indicator for treatment response in metastatic cancer.
Jacot W et al., 2019 [117]	E542K, E545K, H1047R	Metastatic breast cancer	Evaluating prognostic value of *PIK3CA* mutation detection in first-line hormone therapy-treated metastatic breast cancer.	Persistence of a detectable mutation in plasma at 4 weeks of the aromatase inhibitor therapy was correlated with shorter, worse progression-free survival.
Darrigues et al., 2021 [118]	E542K, E545K, H179R H1047R, H1047L and TP53 and AKT1 mutations	ER+/ HER2- metastatic breast cancer.	Assessment of early changes of ctDNA levels in association with palbociclib plus fulvestrant efficacy.	Serial ctDNA analysis is useful for monitoring palbociclib and fulvestrant efficacy before radiological evaluation, and early ctDNA change (e.g., at day 30 of treatment) is a prognostic factor of progression-free survival.
Hrebien S et al., 2019 [119]	E542K E545K H1047R H1047L N345K	ER+ metastatic breast cancer.	Assessment of ctDNA as a predictor of progression-free survival (PFS) and drug efficacy in the BEECH study (paclitaxel plus placebo versus paclitaxel plus AKT inhibitor capivasertib).	ctDNA clearance at week 4 of treatment initiation was identified as the optimal time point to predict PFS.
Rothe et al., 2019 [120]	E545K H1047R H1047L N345K G1049R T1052K K733R and *TP53* mutations	HER2 amplified breast cancer	To evaluate whether ctDNA is associated with response to anti-HER2-targeted therapy in neoadjuvant setting in the NeoALTTO trial.	Mutation detection before neoadjuvant anti-HER2 therapies is associated with decreased pathological complete response.
Sabatier et al., 2022 [121]	R88Q, E542K, E545K, H1047L, H1047R, and *TP53* and *AKT1* mutations	HER2 negative metastatic breast cancer	ctDNA as surrogate marker of treatment efficacy within the phase IB/II TAKTIC trial in which patients received dual AKT and p70 ribosomal protein S6 kinase inhibitor in combination with paclitaxel.	Progression-free survival at 6 months 92% for mutation negative patients and 68% for mutation positive cases at baseline.
Moynahan et al., 2017) [122]	H1047R, E545K, E542K	HR+, HER2− advanced breast cancer	Impact of *PIK3CA* mutations on the efficacy of everolimus in BOLERO-2 study.	Survival benefit by everolimus was independent of *PIK3CA* genotypes.

Rothe et al. evaluated the impact of *PIK3CA* and *TP53* mutations in the response to anti-HER2-targeted therapy in the NeoALTTO trial including 455 patients. *PIK3CA* and/or *TP53* mutations were detected in 31% of baseline tumor samples. Of 69 patients with available ctDNA results at baseline, mutation frequencies were 41%, 20%, and 5% of patients before NAC, at week 2, and before surgery, respectively. ctDNA detection before neoadjuvant therapy was associated with a decreased rate of pathological complete response (pCR) (OR = 0.15; *p* = 0.0089). Interestingly, highest pCR rates were reached in the patients with tumors enriched in HER2 and no detectable baseline mutations. In contrast, women with persistent mutation detection at baseline and week 2 had the poorest response rates. It is concluded that ctDNA detection before neoadjuvant anti-HER2 therapies is helpful in predicting decreased response [120].

Sabatier et al. aimed to assess ctDNA as a marker of treatment efficacy in HER2-negative advanced BCa within the phase IB/II TAKTIC trial in which patients received dual AKT and p70 ribosomal protein S6 kinase (p70S6K) inhibitor in combination with paclitaxel. Plasma samples from some patients were used to evaluate the association of mutational fraction at baseline and after 7 weeks of treatment to PFS and the overall response rate by whole-genome sequencing and ddPCR. All patients with *PI3KCA*, *AKT1,* or *TP53* mutations in tumor had at least one of these variants detectable in plasma. Plasma tumor fraction at baseline was correlated with PFS, with 6 months PFS of 92% for mutation negative patients vs. 68% for mutation positive cases. ctDNA status at week 7 was informative on prognosis. The authors concluded that plasma-based ctDNA analysis may be useful in identifying alterations contributing to resistance development to therapy [121]. The impact of *PIK3CA* hotspot mutations (H1047R, E545K, and E542K) was also assessed on the everolimus efficacy, an mTOR inhibitor, in the BOLERO-2 trial by ddPCR. Survival benefit by everolimus was found to be independent of the type of *PIK3CA* mutations [122].

### 7.3. ddPCR-Based Detection and Clinical Use of Circulating Estrogen Receptor 1 (ESR1) Mutations in Breast Cancer

Although most patients with HR+ breast cancers benefit from first-line endocrine therapy, many of them eventually become endocrine-resistant [123]. The basic mechanism of endocrine resistance is mutations in the ligand-binding domain of estrogen receptor 1 (*ESR1*). These mutations result in constitutive activation of ESR1, even in absence of its ligand, which leads to resistance against endocrine therapy and eventually tumor growth. In primary tumors, *ESR1* mutation rate is low (~1%), but it is more frequent (10–50%) in metastatic, endocrine therapy-resistant BCa and is associated with poor survival. Research efforts in the past decade have focused on biochemical and molecular effects of *ESR1* mutations and the selection of appropriate treatment options [124]. For example, there are indications that patients with HR-positive BCa and emergent *ESR1* mutation during treatment with aromatase inhibitor (AI) may benefit from an early switch to a combination of fulvestrant and palbociclib [125].

Using targeted sequencing, *ESR1* mutations were detected in approx. 15–30% of patients with BCa depending on tumor burden [53,54,126]. Based on the clinical impact of *ESR1* mutations in conferring resistance to endocrine therapy, several studies have to date evaluated *ESR1* mutations in cfDNA in patients with primary or metastatic breast cancers or during resistance to endocrine-therapy by ddPCR [127,128,129,130,131,132,133,134,135] (Table 2). Wang et al. determined the frequency of *ESR1* mutations (K303R, S463P, Y537C, Y537N, Y537S, D538G) by ddPCR in primary and metastatic breast cancer and in cfDNA. Blood samples from four patients were used for the serial monitoring of *ESR1* mutation status. Seven percent of primary ER+ tumors were positive for *ESR1* mutations with very low allele frequencies (0.07% to 0.2%), whereas allele frequency in brain metastases was much higher (34.3–44.9%). Plasma E*SR1* mutations were detected in 7 out of 29 metastatic patients. Interestingly, cfDNA mutation frequency was overall higher compared to primary tumors. Treatment led to changes in *ESR1* mutation detection and allele frequency [127].

E380Q mutation of E*SR1* is responsible for estradiol hypersensitivity and increased DNA binding to the estrogen response element. In a cohort of mBCa patients, the frequency of E380Q mutation was compared with the other *ESR1* mutations (Y537S, Y537N, Y537C, and D538G) in tumor tissue and plasma. In 21% of metastatic tumors, *ESR1* mutations were detected, whereas the E380Q mutation was not detectable in plasma. However, in 46.2% of patients under treatment, increasing levels of other *ESR1* mutations were obvious. Authors concluded that distinct populations of *ESR1* mutations in metastatic tissue and plasma emerge and each *ESR1* mutation may be associated with distinct clinical outcomes [129]. Desmedt et al. compared *ESR1* mutations in tumor and ctDNA between patients with invasive lobular BCa and those with invasive ductal BCa found no difference in terms of mutation frequency and type [134]. Urso et al. aimed to evaluate the concordance between *ESR1* mutation status in metastatic tumors and matched ctDNA in patients with HR+/HER2- BCa. Metastatic tumor biopsy (FFPE DNA) and plasma samples at the progression were available for analyzing the *ESR1* mutations Y537S, Y537C, Y537N, D538G, and E380Q with ddPCR. The concordance between ESR1 status on tumor tissue and plasma was 91% [132].

Schiavon et al. investigated the clinical utility of *ESR1* mutations in ctDNA in patients with advanced BCa. *ESR1* mutations were found exclusively in patients with ER+ BCa exposed to AI, while *ESR1-*positive patients displayed significantly shorter PFS on subsequent AI-based therapy. The timing of first exposure to AI therapy determined the prevalence of *ESR1* mutations: mutation rates were 5.8% vs 36.4 for patients who received endocrine therapy during the adjuvant or metastasis settings, respectively. This shows that *ESR1* mutations rarely arise during adjuvant endocrine therapy, while therapy for metastatic disease selects for mutations. Mechanisms of resistance to AI-based targeted therapy appear to differ between the treatment of micrometastatic and overt metastatic BCa [135].

Najim et al. assessed the frequency of plasma *ESR1* mutations during 5 years of adjuvant hormonal therapy of primary disease and disease recurrence or metastasis during or after termination of endocrine therapy. Tamoxifen was an adjuvant endocrine therapy for primary disease for 57% (12 of 21) of recurrent patients, of which 8 patients had received AI after two years, while for 43% of patients AI was a first-line adjuvant hormonal therapy. The seven most common *ESR1* mutations (E380Q, Y537C, D538G, L536R, S463R, Y537S, and Y537N) were assessed in cfDNA from 21 patients with recurrent breast cancer patients, and any mutation was found in 19% of women [130]. Within the BOLERO-2 double-blind phase 3 study including 189 centers in 24 countries, Chandarlapaty et al. assessed the prevalence of two *ESR1* mutations (Y537S and D538G) in ER-positive mBCa and their prognostic value. The study included postmenopausal women with a diagnosis of mBCa and prior exposure to an AI. mBCa patients were randomized to receive either exemestane plus placebo or exemestane plus everolimus. Baseline plasma samples were available in 541 patients out of 724 (74.7%). Of 541 with available baseline plasma samples, 156 (28.8%) had *ESR1* mutation D538G (21.1%) and/or Y537S (13.3%), and 30 had both. These mutations were associated with shorter OS (wild type: 32.1 months; D538G: 25.99 months; Y537S: 19.98 months; both mutations: 15.15 months) and with more aggressive disease [128]. The findings of this study suggest that each *ESR1* mutation may have different clinical impact. Jeannot et al. developed a multiplex ddPCR assay for E380Q, L536R, Y537C, Y537N, Y537S, and D538G mutations, which displayed a limit of detection ranging from 0.07 to 0.19% in MAF. *ESR1* mutations were identified in plasma of 29% of patients with AI-resistant mBCa. They further analyzed the usefulness of monitoring *ESR1* mutant status in predicting response to fulvestrant (a selective estrogen receptor degrader) and palbociclib (a CDK4/6 inhibitor) therapy in this cohort. The *ESR1* mutational status detected at baseline (both mutant or wild type) had no impact on PFS. At day 30, the majority of patients whose disease progressed in early stages had elevated levels of ctDNA, while decreased or stable ctDNA levels were found in most patients with extended PFS, indicating that mutation detection after 30 days of palbociclib–fulvestrant treatment is a valuable biomarker for predicting PFS [130]. In the two phase III randomized trials (EFECT and SoFEA trials), the clinical utility of baseline *ESR1* mutation analysis was assessed in patients receiving fulvestrant or exemestane [136]. In these trials, patients with HR+ mBCa who had progressed on prior nonsteroidal AI therapy were randomized between fulvestrant and exemestane with the primary objective of assessing the impact of *ESR1* mutation status on PFS and OS. In baseline plasma samples, *ESR1* mutations were found in 30% of patients. In patients harboring *ESR1* mutation, PFS was 2.4 and 3.9 months for exemestane and fulvestrant, respectively. In patients with no mutations, PFS was longer for these agents (4.8 and 4.1 months, respectively). In patients with *ESR1* mutation, 1-year OS rates were 62% and 80% for exemestane and fulvestrant, respectively. For patients without *ESR1* mutations, OS rates were 79% and 81%, respectively. For patients treated with exemestane vs. fulvestrant, detection of plasma *ESR1* mutations at baseline is associated with lower PFS and OS rates [136]. Sim SH et al. [137] evaluated *ESR1* and *PIK3CA* mutations in cfDNA using ddPCR for the efficacy of endocrine therapy in HR+ mBCa patients. *ESR1* mutations (E380Q, Y537N, Y537S, and D538G) and *PIK3CA* mutations (H1047R, E545K, and E542K) were assessed. Of 75 patients, 41.3% were treated with letrozole plus palbociclib, and 37.3% were exposed to exemestane and everolimus. *ESR1* mutations were detected in nearly half (48%) of patients. In the total cohort, the increased number of *ESR1* mutations was informative of shorter time to progression (TTP) of the first endocrine therapy after enrollment (*p*< 0.001). *PIK3CA* mutations were also shown to significantly associate with shorter TTP. In contrast, in patients receiving everolimus treatment, longer TTP rates were found for those with *PIK3CA* mutations. These results reveal that E*SR1* and *PIK3CA* mutations in plasma are associated with clinical efficacy of endocrine therapy in HR+ mBCa patients [137]. Another study [133] was a phase 3 trial (PADA1) in which mBCa patients who were treated with an AI and palbociclib were screened every 2 months for *ESR1* mutations in cfDNA using ddPCR. Of 12,525 available samples, 92% were *ESR1* mutation negative, and a total of 267 patients newly displayed *ESR1* mutations, with a median copy number of 14/mL (ranging from 4 to 225) and a median MAF of 0.83% (0.11–35). Many patients had persistent blood *ESR1* mutations.

Sunderesan et al. investigated *ESR1* mutations in both CTCs and in cfDNA in parallel with the aim of evaluating their impact on endocrine therapy resistance. *ESR1* sequencing from CTCs in 55 women with HR+ mBCa revealed single and multiple mutations in 22% of patients. Using multiplex ddPCR for L536R, Y537S, Y537N, Y537C, and D538G mutations, any *ESR1* mutation in plasma was detected in 17% of patients. A concordance rate of 95% in *ESR1* mutation status was identified from matched CTC and ctDNA samples. Emergence of *ESR1* mutations was shown to correlate with time to metastatic relapse and duration of AI therapy. Furthermore, any *ESR1* mutation was indicative of notable shorter PFS on AI-based therapies [138]. 

### 7.4. ddPCR-Based Detection of TP53 in ctDNA in Breast Cancer

*TP53* is one of most mutated genes in BCa, as demonstrated by targeted sequencing [54,139,140]. The rate of *TP53* mutation frequency in cfDNA varies from 31% to about 50% across studies [141,142]. On the other hand, the reported VAF of *TP53* mutations in plasma have a broader variation than mutation rate, ranging from 0.09% to 70% across different reports [143]. A meta-analysis which reported a mutation rate of 37.8% for *TP53* in cfDNA independent of tumor stage indicated that *TP53* mutations were associated with recurrence, short disease-free survival (DFS), and PFS. However, these findings had no diagnostic power (i.e., low diagnostic accuracy) [144]. A further meta-analysis revealed that *TP53* mutations in cfDNA harbor a diagnostic performance of 0.94 (AUC) in advanced BCa [145]. 

As described above, few studies investigated *TP53* mutations in plasma of BCa patients using ddPCR, doing so mainly in combination with *PIK3CA* [118,120,121]. In the work of Darrigues et al. R175H and H179R mutations of *TP53* along with *PIK3CA* and *AKT1* hotspot mutations were used for serial monitoring of mBCa patients receiving palbociclib and fulvestrant. Of 25 patients with any mutation testable in plasma, 2 had *TP53* mutations [118]. In the NeoALTTO trial on HER2-amplified BCa, plasma *TP53* mutations were detected in 24% of patients with *TP53* mutations in tumors. ctDNA detection (e.g., *PIK3CA* and *TP53* mutations) before NAC was found to be associated with decreased pCR rates [120]. In the phase IB/II TAKTIC trial in which AKT and p70 ribosomal protein S6 kinase inhibitor was combined with paclitaxel in patients with HER2-negative BCa, ctDNA detection—also including TP53 mutations by ddPCR at baseline—was predictive of shorter PFS [121].

*TP53* mutations are detected in up to 80% of TNBC tissues [146]. In patients with TNBC the response to NAC has prognostic value. Riva et al. [147] utilized tracking *TP53* mutations in ctDNA in evaluating the response of TNBC patients to NAC by ddPCR. They detected ctDNA-based *TP53* mutations in 75% of patients at baseline, and the presence of mutations was associated with Ki67 proliferation index and tumor grade and stage. During treatment, a decrease in ctDNA levels was found in nearly all patients, and no ctDNA was detectable after surgery. 

It must be considered that p53 mutations also appear during clonal hematopoiesis of indeterminate potential (CHIP). This means that p53 mutations can also be found in blood plasma irrespective of the presence of cancer disease and account for a number of false-positive results. However, in case of an already diagnosed breast cancer, it has predictive and prognostic value for the outcome of the patients [148].

## 8. Conclusions

Breast cancer is a complex disease with many distinct molecular mechanisms contributing to malignant development and providing the basis for targeted therapies. The use of ctDNA as a non-invasive method may help to overcome many restrictions inherent to tissue biopsy which, as an invasive procedure does not allow the serial monitoring of systemic or targeted therapies or predicting early recurrence and may also not represent the molecular heterogeneity in tumors. The application of ctDNA analysis in BCa opens a spectrum of opportunities that encompass various clinical disease situations from early diagnosis through the detection of minimal residual disease, the early detection of relapse, and the monitoring of treatment. For the purpose of detecting and accurately measuring a very small number of ctDNA molecules in biospecimens, ddPCR remains one of the most powerful technologies. In addition to the detection and absolute determination of hotspot mutations in ctDNA, and low-frequency mutations in particular, ddPCR technology is also used to detect various other cancer-specific signals in cfDNA (e.g., DNA methylation, copy number changes and genetic rearrangements) in various types of clinical biospecimens. While there are several commercial ddPCR assays and technologies on the market, proper validation before use in clinical settings—including analytical sensitivity, imprecision, and method comparison as well as regular internal and external quality controls—are required to guarantee high level cfDNA diagnostics as it is needed for responsible guidance of the patients through the course of cancer disease. In comparison to most sequencing methods, the experimental workflows of ddPCR are much simpler; runtime is much less; and data analysis is less arduous, with minimal complex bioinformatics, and often demonstrates superior sensitivity. ddPCR has been widely used to detect and evaluate the clinical utility of *HER2* amplification and mutations in *PIK3CA*, *ESR1*, and *TP53* genes in plasma of BCa patients. Research shows a relatively high concordance between ddPCR HER2 ctDNA measurements and IHC/FISH measurements in tissue, highlighting the potential of ddPCR to serve as a useful method in the companion diagnostics toolbox as it relates to the assessment of HER2 amplification status and the serial monitoring of patients on anti-HER2 therapies. As *PIK3CA* mutations are frequently detected in BCa, targeting of *PIK3CA* in HR+/HER2− mBC has demonstrated remarkable benefits following the development of resistance to endocrine therapy, and these alterations have predictive value in the response to various PI3K pathway inhibitors. Several studies were conducted to determine the frequency and prognostic value of *PIK3CA* mutations in blood circulation of BCa patients using ddPCR, and many of these demonstrated its usefulness in serial monitoring and the prediction of the efficacy of endocrine and/or targeted therapies. Acquisition of *ESR1* mutations conferring treatment resistance to AIs affects therapeutic efficacy of hormonal therapies in ER-positive BCa. As *ESR1* mutations are detected in 15–30% of breast tumors, several studies evaluated hotspot *ESR1* mutations using ddPCR in the plasma of patients with primary or metastatic breast cancers or with resistance to endocrine therapy. It seems that distinct populations of *ESR1* mutations in metastatic tissue and plasma may have different clinical significance. *ESR1* mutations detected at baseline or in AI-resistance are associated with poor survival, and emergence of or increases in *ESR1* mutations were found to be indicative of progression/metastatic relapse under endocrine therapy. As a defined panel of mutations in known genes is informative as predictive, prognostic or monitoring markers in early and advanced BC, non-invasive ddPCR-based diagnostic in plasma cfDNA is a useful tool for better guidance of patients during the course of BC disease.

## Figures and Tables

**Table 2:** An overview on the reports which analyzed plasma ESR1 mutations in breast cancer patients using droplet digital PCR.

Reference	ESR1 Mutations Analyzed	Study Cohort	Goal of the Study	Main Finding
Wang P et al., 2016 [127]	K303R, S463P, Y537C, Y537N, Y537S, D538G	ER+ primary or metastatic breast cancers	Determining the *ESR1* mutation frequency in primary and metastatic breast cancer and in cfDNA.	*ESR1* allele frequencies in brain metastases and cfDNA were higher than in primary tumors. Endocrine therapy was associated with *ESR1* mutations.
Takeshita et al., 2017 [129]	E380Q, Y537S, Y537N, Y537C, D538G	Metastatic breast cancer	Assessing the E380Q mutation in comparison with the other *ESR1* mutations in tumor and plasma.	Distinct populations of *ESR1* mutations in metastatic tissue and plasma. Emergence of many mutations in plasma during therapy, with each *ESR1* mutation having a different clinical significance.
Desmedt C et al., 2019) [134]	E380Q, Y537S/C/, D538G	Metastatic invasive lobular breast cancer and invasive ductal breast cancer	Comparative analysis of *ESR1* mutations in tumor (MSKCC-IMPACT trial) and ctDNA (SoFEA and PALOMA-3 trials) between invasive lobular breast cancer and invasive ductal breast cancer.	Invasive lobular breast cancer and invasive ductal breast cancer did not differ in terms of frequency and type of ESR1 mutations.
Urso L et al., 2021 [132]	Y537S, Y537C, Y537N, D538G, E380Q	HR+/HER2-negative metastatic disease	Evaluating the concordance between ESR1 status in metastatic tumors and matched ctDNA at progression.	High concordance (91%) between ESR1 status on tumor tissue and cfDNA.
Schiavon et al., 2015 [135]	L536R, Y537S, Y537N, Y537C, D538G	Advanced breast cancer	Assessing the clinical relevance of *ESR1* mutations.	*ESR1* mutations were detected exclusively in patients exposed to aromatase inhibitor and associated with shorter PFS. *ESR1* mutations are selected during therapy for metastatic disease.
Najim et al., 2019 [130]	E380Q, Y537C, D538G, L536R, S463R, Y537S, Y537N	ER positive recurrent BCa	Determining the frequency of *ESR1* mutations in recurrent BCa.	Any *ESR1* mutation was found in 19% of patients with recurrence or progression on hormonal therapy.
Chandarlapaty et al., 2016 [128]	Y537S, D538G	Postmenopausal ER+ metastatic breast cancer with an prior exposure to aromatase inhibitor	Evaluating prognostic significance of *ESR1* mutations within the BOLERO-2 double-blind phase 3 study (exemestane plus placebo or exemestane plus everolimus)	*ESR1* mutations were associated with shorter overall survival and with more aggressive disease
Jeannot et al., 2020 [131]	E380Q, L536R, Y537C, Y537N, Y537S, D538G	Aromatase-inhibitor resistant metastatic breast cancer	Evaluating clinical benefit of monitoring of ESR1 mutations during Fulvestrant- Palbociclib treatment.	*ESR1* mutations were identified in plasma of 29% of patients progressed under aromatase inhibitor. Mutation monitoring predicts the clinical benefit from palbociclib–fulvestrant.
Turner et al., 2020 [136]	Multiplex 1 E380Q, L536R, Y537C, D538G Multiplex 2 S463P, Y537N, Y537S.	Patients with HR+ metastatic breast cancer patients who had progressed on prior aromatase inhibitors	Assessing impact of *ESR1* mutation status on progression-free (PFS) and overall survival (OS) in therapy with fulvestrant vs. exemestane.	Detection of *ESR1* mutations in baseline ctDNA is associated with poor prognosis in patients treated with exemestane vs. fulvestrant.
Sim SH et al., 2021 [137]	E380Q, Y537N, Y537S, D538G and PIK3CA (H1047R, E545K, and E542K)	HR+ metastatic breast cancer patients	Impact of *ESR1* mutation detection in therapy with letrozole with palbociclib vs. exemestane and everolimus.	Increasing numbers of *ESR1* mutations are associated with time to progression of the first endocrine therapy.
Callens et al., 2022 [133]	Exon 5 and 8 mutations	Metastatic breast cancer patients	Screening for activating *ESR1* mutations every 2 months during aramatose inhibitor and palbociclib within the phase 3 trial (PADA1).	A total of 267 patients newly displayed *ESR1* mutations, and 648 samples (20% patients/5% samples) displayed persistent *ESR1* mutations. Feasibility and accuracy of ESR1 mutation tracking by ddPCR for therapeutic interventions.
Sunderesan et al., 2021 [138]	L536R, Y537S, Y537N, Y537C, D538G	HR+ metastatic breast cancer	Impact of *ESR1* mutations in circulating tumor cells and plasma for the determination of endocrine resistance.	Emergence of *ESR1* mutations in recurrent patients was correlated both with time to relapse and duration of endocrine therapy. *ESR1* mutation was associated with shorter survival on therapy with aramatose inhibitor.

## Data Availability

Not applicable.
